# Oxidative Stress in Aortic Valves Associated with Infective Endocarditis: A Report on Three Cases

**DOI:** 10.3390/diagnostics14242807

**Published:** 2024-12-13

**Authors:** María Elena Soto, Linaloe Manzano-Pech, Verónica Guarner-Lans, Hugo Rodríguez-Zanella, Israel Pérez-Torres, Elizabeth Soria-Castro

**Affiliations:** 1 Research Direction, Instituto Nacional de Cardiología Ignacio Chávez, Juan Badiano 1, Sección XVI, Tlalpan, México City 14080, Mexico; elena.soto@cardiologia.org.mx; 2 Cardiovascular Line American British Cowdray Medical Center, Sur 136 Numero 116 Col Álvaro Obregón, México City 01120, Mexico; 3 Department of Cardiovascular Biomedicine, Instituto Nacional de Cardiología Ignacio Chávez, Juan Badiano 1, Sección XVI, Tlalpan, México City 14080, Mexico; loe_mana@hotmail.com; 4 Department of Physiology, Instituto Nacional de Cardiología Ignacio Chávez, Juan Badiano 1, Sección XVI, Tlalpan, México City 14080, Mexico; veronica.guarner@cardiologia.org.mx; 5 Department of Echocardiography, Instituto Nacional de Cardiología Ignacio Chávez, Juan Badiano 1, Sección XVI, Tlalpan, México City 14080, Mexico; drzanella@gmail.com

**Keywords:** infective endocarditis, oxidative stress, cardiac valve, antioxidant enzymes, redox homeostasis

## Abstract

**Background/Objectives:** Infective endocarditis (IE) most commonly results from infections by Gram-positive bacteria, and, in this condition, the redox homeostasis is lost due to the overproduction of H_2_O_2_, leading to the overstimulation of the immune system and the upregulation of the production of proinflammatory cytokines. The aim of this study was to evaluate the levels of oxidative biomarkers and the enzymatic and non-enzymatic antioxidant systems in subjects with IE. **Methods:** The study included three cases with IE that had undergone aortic valve replacement (AVR) surgery that was complicated by IE, comparing them with subjects with AVR without IE. We determined the malondialdehyde (MDA), total antioxidant capacity (TAC), carbonyl group concentration, glutathione (GSH), thiols and the nitrate/nitrite ratio (NO_3_^−^/NO_2_^−^) in homogenized tissue from the cardiac valves. We also measured the activity of glutathione-S-transferase (GST), glutathione peroxidase (GPx), glutathione reductase (GR) and thioredoxin reductase (TrxR). The superoxide dismutase (SOD) isoforms and peroxidase activity were determined using native gels. **Results:** There were increases in the activity of antioxidant enzymes such as GST, SOD isoforms and peroxidases (*p* ≤ 0.01) and decreases in oxidative stress markers such as GSH (*p* = 0.05); meanwhile, MDA and carbonylation were increased (*p* ≤ 0.05). **Conclusions:** The results suggest that bacterial infections favor oxidative stress in the aortic valves, which increases the SOD isoforms and peroxidase activity. This contributes to the loss of the intricate redox homeostasis system in patients with IE, causing a positive feedback loop in the oxidative background that results in damage to the heart, likely leading to a fatal outcome.

## 1. Introduction

Gram-positive bacteria such as *Streptococcus* and *Staphylococcus* are the most common bacteria responsible for infective endocarditis (IE). These bacteria constitute 75% of the isolated microorganisms in this condition, but other species have also been found, such as *Escherichia coli* and *Pseudomonas aeruginosa*, among others [1]. The intracardiac site that is most frequently compromised is the aortic valve. The clinical characteristics present in patients with IE are fever, sepsis, septic shock, heart failure, chest pain when breathing and fatigue. The damage to the heart results from the formation of vegetations, which are composed of masses of infectious organisms, fibrin and platelets that are held together by agglutinating antibodies produced by the bacteria [2]. Pharmacological treatment may include aminopenicillins, cephalosporins and penicillin [3].

Gram-positive bacteria contain lipopolysaccharides (LPSs) in their cell walls, which are responsible for the overexpression of the host’s immune system, the induction of a cascade of proinflammation caused by cytokines and adhesion to epithelial cells. These bacteria also have powerful toxic effects [4]. The overstimulation of the immune system is mainly due to the activation of macrophages, monocytes and neutrophils, and there is increased secretion of proinflammatory cytokines such as interleukin (IL)-1, -6 and -8, which can, in turn, upset the redox homeostasis in the body. This homeostasis includes the enzymatic and non-enzymatic antioxidant systems [5]. The lost redox homeostasis in IE is characterized by an increase in the superoxide anion (O_2_^–^), mainly derived from nicotinamide adenine dinucleotide phosphate (NADPH) oxidase and mitochondrial dysfunction. This anion contributes to increases in other reactive oxygen species (ROS), such as hydrogen peroxide (H_2_O_2_) [6]. ROS may also lead to the production of hydroxyl radicals (OH^–^), which induces peroxidative damage to proteins, lipids, carbohydrates and nucleic acids in cardiomyocytes. This is due to the large amount of iron present in cardiomyocytes, which is involved in the Fenton and Haber–Weiss reactions in the presence of OH^–^. In this sense, there is an association between the generation of myocardial ROS and lipid peroxidation (LPO) and the contractile dysfunction of the left ventricle [7]. Therefore, against this oxidative background, an alteration in the antioxidant systems may be expected, but, so far, the degree of participation of each particular antioxidant system remains unknown.

On the other hand, the clinical management of IE often requires surgical intervention or highly complex invasive procedures that are associated with several diagnostic and therapeutic dilemmas. These dilemmas are due to the lack of experience of the clinicians and to the lack of published data and guidelines on the appropriate treatment [8]. Therefore, the mortality rates from this condition are high, and this constitutes a challenge in need of surveillance proposals. The existence of therapeutic guidelines for treatment may be improved by implementing preventive and therapeutic management [9]. Therefore, research on the role played by the deregulation of the redox homeostasis within the infectious process is relevant, regardless of the type of triggering agent in the disease. An understanding of how redox homeostasis is compromised and its association with the process of bacterial infection could help to ensure better outcomes for patients. It would therefore be of importance to propose an adjuvant therapy that could contribute to reestablishing redox homeostasis. Therefore, the aim this preliminary study on three subjects with IE was to evaluate the levels of oxidative stress (OS) markers and the enzymatic and non-enzymatic antioxidant systems in their native or prosthetic aortic valves that required aortic valve replacement (AVR) or mitral valve replacement because of IE, in comparison with three subjects who also underwent AVR but without developing endocarditis.

## 2. Materials and Methods

### 2.1. Recruited Patients with IE and Control Subjects

We performed a preliminary analytical retrospective review of the medical records of a list of patients diagnosed as having IE according to the modified Duke/ESC 2015 clinical criteria (protocol with identifier code CI-035-2024) [10]. The three selected cases with IE had been discussed by a team of expert surgeons, interventionists and clinicians, who had agreed that they required surgery and transcatheter aortic valve implantation (TAVI). The patients had given their informed consent prior to surgery. The patients had also agreed that their tissue could be used for microbiological and histopathological studies. In summary, the cases had received a clinically documented diagnosis, which was supported by evidence, a positive bacterial culture and imaging. Aortic and valve tissue was obtained during the valvuloplasty surgery because of the presence of endocarditis. Samples were obtained from the region proximal to the lesion by vegetation, which could be present in different segments. The sample of each tissue was sectioned into two portions, placed in tubes under liquid nitrogen and frozen at −70 °C until studied. The control subjects (CSs) were enrolled for the surgery to substitute valves for mechanical prosthesis. The first case underwent AVR; the second case was implanted with a mechanical valve; and the third case underwent mechanical St. Jude AVR. These patients were selected because of the valve replacement that they underwent, which was similar to that of the subjects with IE. Since they were not healthy subjects, it was expected that they also had alterations in redox homeostasis. The difference lay in the IE present in the patients who did have the bacterial infection, against those that did not have it.

### 2.2. Echocardiographic Study

Transesophageal echocardiography was performed using an X7-2t transducer, 2–7 mHz (Phillips Fort Myers, Florida 6391 ARC Way, 33966, Fort Myers, FL, USA). All patients underwent a comprehensive study due to the high clinical suspicion of endocarditis under sedation.

### 2.3. Cardiac Valve Homogenization

The segment from the aortic valve was homogenized under liquid nitrogen after adding KH_2_PO_4_ (2 mL) 0.05 mM, pH 7.3, in the presence of 20 µL antiprotease inhibitors (2 μM leupeptin, 2 μM pepstatin A, 0.1% aprotinin and 1 mM PMSF). The method of Lowry was utilized to determine the protein concentration in the homogenates [11]. Biochemical variables and OS markers (except for those in the native gels and Western blot analysis) were determined in duplicate.

### 2.4. Determination of Malondialdehyde

The malondialdehyde (MDA) level was quantified spectrophotometrically at 532 nm. First, 100 μg of homogenized tissue from the aortic valves of the patients with IE and CSs were used. Then, 1 mL of the KH_2_PO_4_ buffer at pH 7.4 plus 100 μL methanol with BHT (4%) were added to the sample and incubated at 37 °C for 30 min. Then, 1.5 mL of 2-thiobarbituric acid (0.8 M) was added and the sample was incubated at 90 °C for 1 h. Afterwards, 1 mL of KCl (5%) and 4 mL of n-butanol were added; the sample was shaken for 30 seg and centrifuged for 2 min at 4000 rpm. The butanol phase was extracted, and the absorbance was measured [12].

### 2.5. Evaluation of TAC

The TAC was quantified spectrophotometrically at 593 nm. First, 100 μg of homogenized tissue from the aortic valves of patients with IE and CSs was taken in the presence of a mixture that contained C_2_H_3_O_2_ at 300 mM, FeCl_3_·6H_2_O at 20 mM, 2,4,6-tris-2-pyridyl-s-triazine at 10 mM and HCl at 40 mM, at pH 3.6 (1.5 mL, at a ratio of 10:1:1 *v*/*v*), and incubated at 37 °C for 15 min, and the absorbance was measured [13].

### 2.6. Carbonylation

To evaluate the carbonyl groups, 100 μg of homogenized tissue from the aortic valve was mixed with 500 μL of HCL (2.5 M), and, in parallel, another sample was mixed with 500 μL of 2,4-dinitrophenylhydrazine and incubated at room temperature for one hour in the dark. Before incubation, 500 μL of C_2_HCl_3_O_2_ at 20% was added and the sample was centrifuged at 15,000× *g* for 5 min. The button was recovered and washed two times; then, 1 mL of C_2_H_5_OH/C_4_H_8_O_2_ was added and the mixture was incubated for 10 min and centrifuged at 15,000× *g* for 10 min. Finally, 1 mL of CH_6_CIN_3_ (6 M) in KH_2_PO_4_ (20 mM) at pH 2.3 was added and the mixture was incubated at 37 °C for 30 min. The absorbance was read at 370 nm [12].

### 2.7. GSH and Thiols

For the determination of GSH, 100 μg of homogenized tissue from the aortic valve was used, and this molecule was detected spectrophotometrically at 412 nm, according to Ellman’s method. The thiol groups were read spectrophotometrically at 415 nm using 50 µg of homogenized tissue from the aortic valves of patients with IE and CSs. The determination was performed according to Erel and Neselioglu’s method [14].

### 2.8. NO_3_^−^/NO_2_^−^ Ratio Determination

To evaluate the NO_3_^−^/NO_2_^−^ ratio, 100 μg of homogenized tissue from the aortic valves of patients with IE and CSs was previously deproteinized with 100 μL of a mixture that contained NaOH (0.5 N) and ZnSO_4_ (10%). Then, 10 μL of cytochrome c reductase (NADPH) (Sigma Aldrich Cat# 24479) was added and the sample was incubated for 30 min at 37 °C. Afterwards, 200 µL of sulfanilamide (1%) and N-naphthyl-ethyldiamine (0.1%) was added and the absorbance was measured at 540 nm [15].

### 2.9. Determination of Antioxidant Enzymes That Employ GSH

To evaluate the activity of glutathione-S-transferase (GST), glutathione peroxidase (GPx), glutathione reductase (GR) and thioredoxin reductase (TrxR), we used 100 μg of homogenized tissue from the aortic valves of patients with IE and CSs [12]. The activity of GR was expressed as μmol of reduced GSSG/min/mg of protein, with an extinction coefficient of 6220 M^−1^ cm^−1^. The activity of GST was expressed as units of GS-TNB mol/min/mg of protein, with an extinction coefficient of 14,150 M^−1^ cm^−1^. The activity of GPx was expressed as nmol of NADPH oxidized/min/mg of protein, with an extinction coefficient of 6220 M^−1^ cm^−1^. The samples were incubated and monitored at 340 nm for 6 min at 37 °C. For the determination of the activity of TrxR, the sample was incubated and monitored at 412 nm for 6 min at 37 °C in the presence or absence of 20 µM auranofin, and the activity was expressed as TNB nmol/min/mL of protein, with an extinction coefficient of 13,600 M^−1^ cm^−1^.

### 2.10. Determination of Superoxide Dismutase Isoforms and Peroxidase Activity

The activity of superoxide dismutase (SOD) isoforms and peroxidase was determined through non-denaturing gel electrophoresis [12]. First, 25 µg of homogenized tissue from the cardiac valves of the IE and CS patients was applied directly to non-denaturing 10% polyacrylamide gels. The electrophoresis was carried out at 120 volts for 4 h. For the activity of the SOD isoforms, the gel was incubated with 25 mL of nitro blue tetrazolium (2.45 mM) for 20 min, and then incubated with 20 mL of a buffer that contained KH_2_PO_4_ (36 mM), EDTA (28 mM) and riboflavin (28 Μm) at pH 7.8 and exposed to UV light for 10 min. Purified SOD from bovine erythrocytes with specific activity of 112 U/mg per protein (Sigma-Aldrich, St. Louis, MO, USA) was used (positive control) to calculate the activity of the enzymes. For the activity of the peroxidases, the gel was washed with distilled water for 5 min, and then it was incubated with 20 mL of 3,3,5,5-tetramethylbenzidine (3 mg/mL) dissolved in CH_3_-OH/CH_3_COOH/H_2_O (1:1:1 *v*/*v*) with H_2_O_2_ (300 μL) for 10 min. Horseradish peroxidase (35 μL) at 178.5 μg was used as a standard. The SOD isoforms and peroxidase activity in the gels were analyzed using densitometry with a Kodak Image^®^ 3.5 system.

### 2.11. Statistical Analysis

Continuous variables are expressed as medians with minimum–maximum ranges. Categorical variables, such as percentages and frequencies, are reported. The Shapiro–Wilk test was used to test the normality of distribution. When there was a Gaussian distribution in the data, non-parametric tests (Mann–Whitney) were used to detect significant independent variables. The graphical results are shown as the median, first quartile, third quartile and half dotted line. Sigma Plot**^®^** version 15 was used to perform the analysis and generate the graphs (Systat Software Inc., San Jose, CA 95131, USA, EE. UU, North First Street, Suite 360, Jandel Corporation, San Jose, CA, USA). Differences were considered statistically significant when *p* ≤ 0.05.

## 3. Results

### 3.1. General Characteristics of the Patients with IE and the CSs

Table 1 describes the general characteristics and serum biochemical parameters of the three patients with IE and of the CSs.

The demographic characteristics and admission conditions of the patients and controls are shown in Table 2. Surgical interventions were performed in two cases of IE, and one underwent TAVI. The three CS cases were treated by surgery.

**Figure 1:**
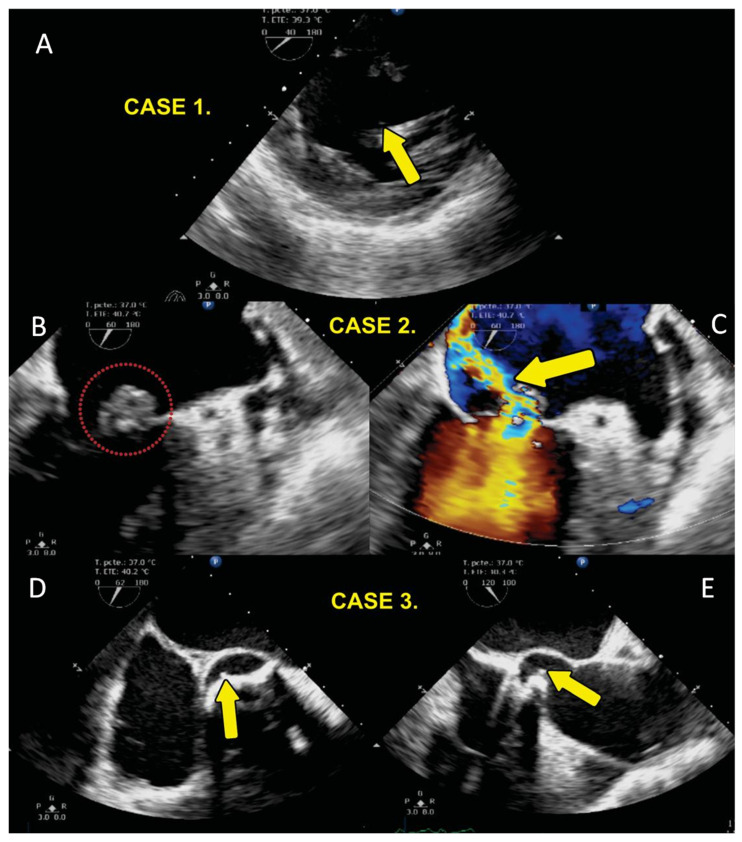
Transesophageal echocardiographic analysis of the patient series. Panel (**A**): case 1. Transgastric short axis showing vegetation (yellow arrow) in relation to the subvalvular mitral apparatus. Case 2 showing mitral valve endocarditis (red circle) in panel (**B**) and severe mitral regurgitation (yellow arrow) in panel (**C**). Case 3. Transesophageal short axis in panel (**D**) and three-chamber view in panel (**E**), showing vegetation in the aortic valve (yellow arrow).

Figure 1 shows the echocardiographic findings in case 1 with IE. A transgastric short axis showing vegetation (yellow arrow) in relation to the subvalvular mitral apparatus is observed. In case 2, the left side shows mitral valve endocarditis (red circle), and the right side is from the same case. It shows severe mitral regurgitation (yellow arrow). In case 3, a transesophageal short axis is shown on the left side, and a three-chamber view, showing vegetation in the aortic valve (on right side, yellow arrow), is observed.

### 3.2. Oxidative Markers

**Figure 2:**
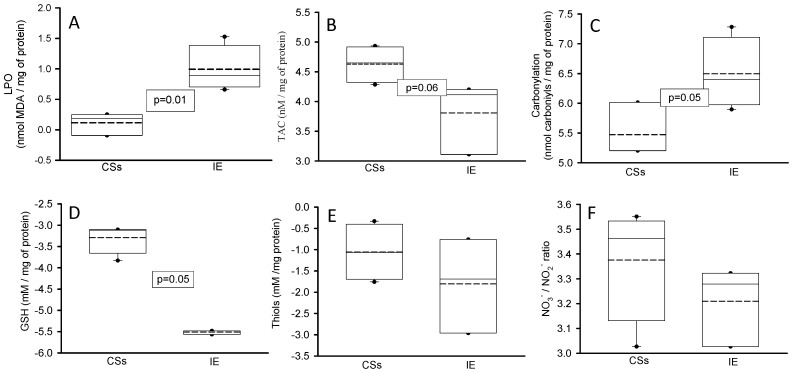
Oxidative stress markers in the homogenized tissue of the cardiac valve from the CSs and patients with IE: MDA (panel (**A**)), TAC (panel (**B**)), carbonylation (panel (**C**)), GSH (panel (**D**)), thiol groups (panel (**E**)) and NO_3_^–^/NO_2_^–^ ratio (panel (**F**)). The values are expressed as the median, first quartile, third quartile and half dotted line. The dark circles that stand out from each bar are the outliers. Abbreviations: CSs = control subjects, IE = infective endocarditis, MDA = malondialdehyde, TAC = total antioxidant capacity, GSH = glutathione, NO_3_^–^/NO_2_^–^ = nitrate and nitrite, LPO = lipoperoxidation.

The OS markers are shown in Figure 2. MDA was increased (*p* = 0.01, panel A) and carbonyl groups showed an increase that was statistically significant (*p* = 0.05, panel C) in the homogenized tissue of the aortic valve in the patients with IE in comparison with the CSs. GSH (*p* = 0.05, panel D) was diminished, However, TAC showed only a tendency to decrease in the patients with IE (*p* = 0.06). The NO_3_^–^/NO_2_^–^ ratio and thiol groups did not show significant changes (panels E and F, respectively) in comparison to the CSs.

### 3.3. Activity of Antioxidant Enzymes That Employ Glutathione

**Figure 3:**
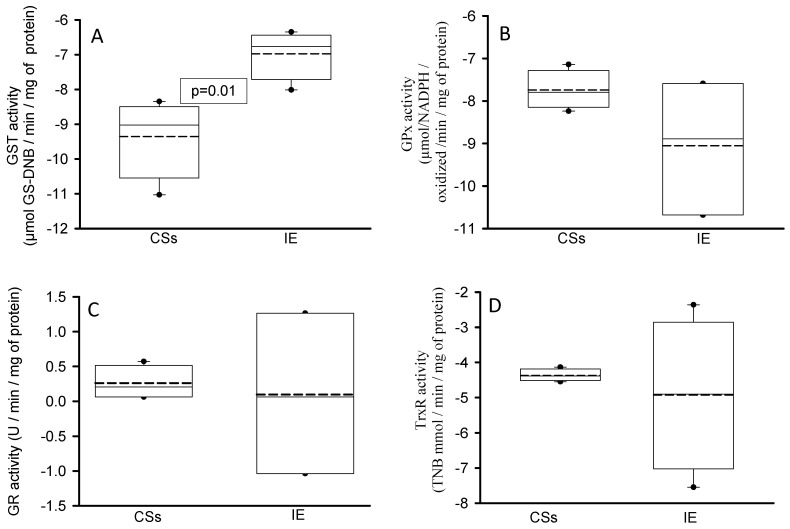
Determination of some antioxidant enzymes that employ GSH, such as GST (panel (**A**)), GPx (panel (**B**)), GR (panel (**C**)) and TrxR (panel (**D**)), in the homogenized tissue of the aortic valve from CSs and patients with IE. The values are expressed as the median, first quartile, third quartile and half dotted line. The dark circles that stand out from each bar are the outliers. Abbreviations: CSs = control subjects, IE = infective endocarditis.

Figure 3 shows that the GST activity in the homogenized tissue of the aortic valve was increased in patients with IE in comparison with the CSs (*p* = 0.01, panel A). However, the activity of GPx, GR and TrxR did not show significant differences between the patients with IE and the CSs (panels B, C and D, respectively).

### 3.4. Peroxidase and SOD Activity

**Figure 4:**
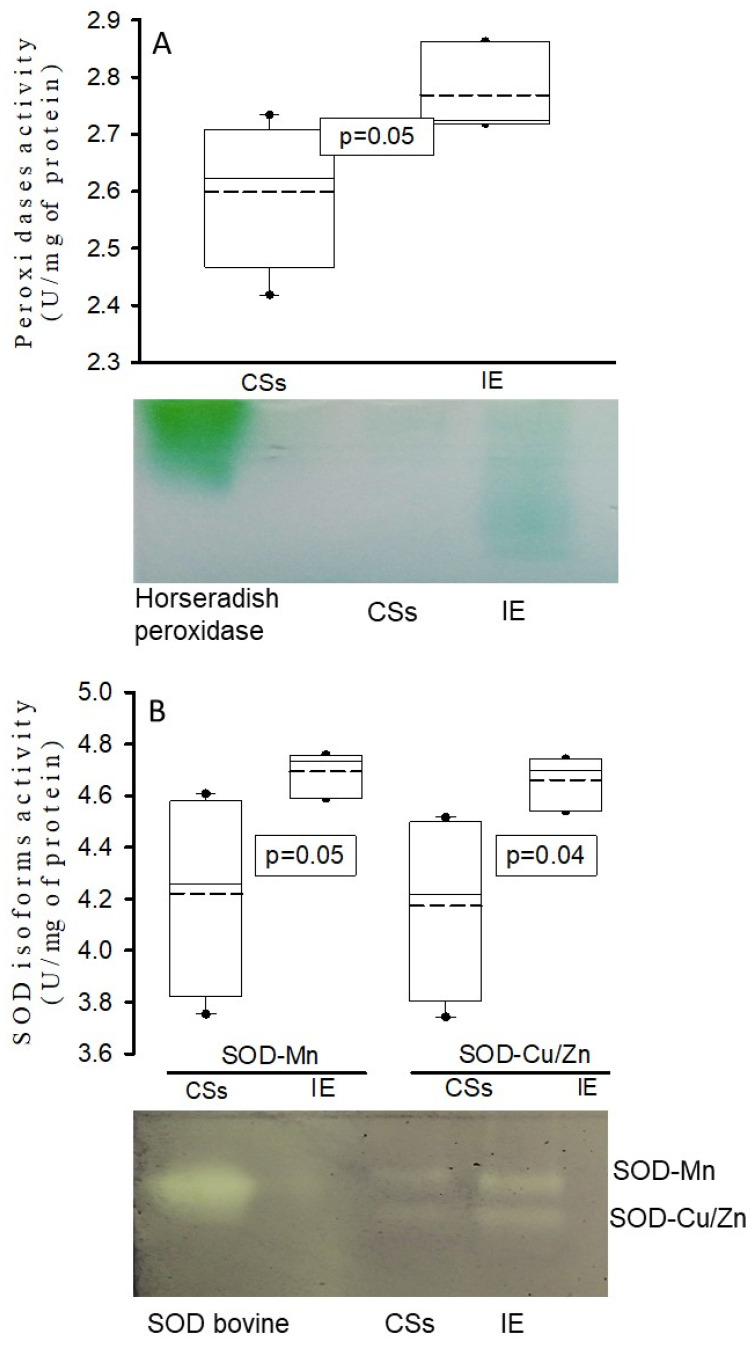
Activity of peroxidases (panel (**A**)) and SOD isoforms (panel (**B**)) in homogenized tissue of the aortic valve. The values are expressed as the median, first quartile, third quartile and half dotted line. The dark circles that stand out from each bar are the outliers. Abbreviations: CSs = control subjects, IE = infective endocarditis, SOD = superoxide dismutase.

The activity of peroxidases (*p* = 0.05) and the SOD isoforms (*p* = 0.05 and *p* = 0.04) were increased in the homogenized tissue of the aortic valve in the IE patients in comparison with CSs (Figure 4A,B, respectively).

## 4. Discussion

In this study, we evaluated the levels of OS markers and the enzymatic and non-enzymatic antioxidant systems in native or prosthetic valves from patients with IE that required AVR and in controls with AVR without IE. We also described the effects of the different therapeutics used and compared the results with those from subjects undergoing AVR without endocarditis.

The loss of redox homeostasis in IE is characterized by the overproduction of O_2_^–^ and H_2_O_2_, which results from the bacterial infection and increases the NADPH activity in the cardiomyocytes of the aortic valve. This increase, in turn, favors the activity of enzymes that are responsible for the detoxification of these ROS, such as the SOD isoforms and the family of peroxidases [6,7]. Our results show that the activity of the SOD isoforms, which have copper/zinc (Cu/Zn) and manganese (Mn) in the catalytic center (SOD-Cu/Zn cytosolic and SOD-Mn mitochondrial, respectively), and which are necessary for the dismutation processes of O_2_^−^ to H_2_O_2_, was increased in the homogenized tissue of the aortic valve in the IE patients. This result was probably due to excess O_2_^–^, which may be provided by the overactivity of NADPH associated with the bacterial infection. However, the activity of the SOD isoforms may favor a decrease in O_2_^−^, which, in turn, induces a high concentration of H_2_O_2_. This elevated concentration is the substrate for other enzymes, such as GPx and the peroxidase family. Our results showed that the GPx activity had a tendency to decrease but without reaching statistical significance. This could be due to the small number of cases, but also to the fact that bacterial infections can decrease the activity of endogenous GPx as a mechanism for survival to perpetuate the infectious process [16]. However, the activity of peroxidases was increased in IE patients. Bacterial infections can activate eosinophil peroxidase and the myeloperoxidases in neutrophils and monocytes [17]. These enzymes, which contain a hemo group in their catalytic centers, that are effective tools against bacterial infections. They utilize H_2_O_2_ to catalyze the oxidation of halides to generate hypohalous acids, which are powerful oxidant agents that are capable of oxidizing membrane lipids, proteins, RNA and DNA from microorganisms. This leads to the killing of bacteria [18]. Our results show that the activity of peroxidases was increased in the homogenized tissue of the cardiac valves in patients with IE. This suggests that the peroxidase activity is increased to counteract the bacterial infection.

In addition, the increase in H_2_O_2_ can favor the formation of OH^−^, which oxidizes the polyunsaturated fatty acids of the cells, forming oxidized lipid peroxides such as MDA, an LPO marker. Our results showed that the level of MDA was increased in the homogenized tissue of the cardiac valve in patients with IE. This increase also favors the loss of TAC. In this sense, the concentration of GSH is depleted in bacterial infections. The GSH reduction in the host can be expected since it is an antioxidant molecule that helps to fight the increase in ROS associated with the infection process. Furthermore, throughout the infection, the bacteria can use the host’s GSH for survival, replication, virulence and other processes. In fact, *Streptococcus* consumes it for nutritional purposes [19]. Our results showed that the GSH concentration was decreased in the homogenized tissue of the cardiac valve in patients with IE, reinforcing the findings mentioned above. Moreover, the loss of GSH favors the oxidant background in patients with IE, as well as an increase in LPO and a decrease in TAC. However, the loss of GSH can also be due in part to its demands. In this sense, the activity of GST, which is an enzyme that employs GSH, forming less toxic glutathione S-conjugates, including products of LPO in the process of detoxification, was increased in the homogenized tissue of the aortic valve. A possible explanation may be that it constitutes a compensatory mechanism to eliminate LPO products such as 4-hydroxy-2-nonenal and bacterial LPS. In this sense, a study in a pediatric population (9–17 years of age) affected by *Pseudomonas aeruginosa* and *Staphylococcus aureus* that were causing chronic lung infections showed an increase in GST [20]. Another study demonstrated that a mixture of GST-mu-class isoforms from *F. hepatica*, administered intraperitoneally 1 h after an LPS injection, was capable of significantly suppressing the LPS-induced cytokine storm in a mouse model of septic shock [21]. Furthermore, the lack of significant changes in some enzymes, such as GR, TxrR and GPx, and oxidative markers such as thiol groups, the NO_3_^–^/NO_2_^–^ ratio and carbonyl groups, could be due to the small number of patients with IE studied.

On the other hand, aortic stenosis is a disease that can occur in several etiologies. It is also often found in the general population with risk factors such as age, obesity and dyslipidemia, among others. It is also widely associated with the presence of bicuspid aortic valves and autoimmune processes. Regardless of the etiological clinical factor, once the subject requires surgery for an aortic valve replacement, their condition can be complicated by the presence of endocarditis. There are still gaps in our knowledge of the mechanisms of this disease regarding its interaction with inflammation, but one of them is OS. When choosing to conduct this preliminary study, we intended to determine the importance of evaluating oxidative deregulation and its significance for future clinical or cohort trials with an appropriate number of patients and sample sizes. Exploratory studies allow for the determination of whether a hypothesis deserves special attention and whether to invest resources in long-term prospective studies when the frequency of the disease is not particularly high.

The patients with IE that participated in this study were carefully chosen for their clinical representativeness. In this sense, in the first patient with endocarditis, it was associated with the presence of autoimmunity. There are few reported cases of this association [22,23]. The second patient did not have an autoimmune condition, and there are large studies of series of patients with similar conditions; however, little information on the participation of the OS process in the mechanism of damage in the infection has been reported. The reports that are available [24] refer to animal models or studies carried out at an experimental level in vitro, but not in human tissue. In the third case, aortic stenosis was performed with interventional therapy, which is most commonly indicated in older subjects with adjacent comorbidities, where the surgical risk is very high [25,26].

Regarding the specific characteristics of each patient, case 1 corresponded to a 55-year-old woman with an established diagnosis of Takayasu arteritis (TA), which developed endocarditis. The culture of the aortic valve indicated the presence of *Staphylococcus aureus* and *epidermidis* (Supplementary Materials Video S1). Therefore, in this case, IE was fully documented. Although there are few reported cases of large-vessel vasculitis, the coexistence of vasculitis and IE may occur [27]. However, there are reports that TA can be confused with IE, especially when the signs and symptoms resemble the inflammatory activity or when multiple aneurysms are found [28]. This case was unfortunate, and the patient died a year after the valve replacement, since, during the post-surgery period, she experienced a complication in the form of a cerebral vascular disease. Although she was treated with timely surgical intervention, comprehensive therapeutic management could improve the results in patients with similar conditions. Cases of TA are rare, and it is difficult to obtain large series with the coexistence of IE. However, it would be important to evaluate the use of antioxidants as an adjuvant therapy in this complex condition, where inflammation persists due to the disease and can be associated with an infectious state and the deregulation of OS. In this study, all patients had high LPO, and there are no previously reported, similar studies. Therefore, this is a case to highlight.

The second patient was a 78-year-old woman who was treated for aortic stenosis with TAVI. At 48 h after this procedure, she presented endocarditis and underwent surgery. This procedure is cutting-edge and IE occurs with a low frequency in the early phase, before 30 days (Supplementary Materials Video S1) [2]. The patient developed IE within the first 48 h of implantation. In most published cohorts, *Enterococcus spp*. have been identified as the most common pathogens, followed by *Staphylococcus aureus*, whose incidence is relatively low, ranging between 0.1 and 3% [29,30]. However, IE post-TAVI is an early complication, and it is associated with a poor prognosis, not only in comparison with native valve endocarditis but also in comparison to surgical valve replacement for endocarditis, regarding both valve dysfunction and patient mortality [2]. The clinical profile considering most TAVI candidates includes elderly patients who are at a high surgical risk due to the presence of several comorbidities at the time of the intervention [31]. This also represents a risk that can enhance common IE pathogens and the presence of other opportunistic bacteria [32]. In this patient, the complication due to IE was associated with the presence of *Enterobacter cloacae* and *Klebsiella oxytoca*. However, it also highlights the moment at which a solution could be implemented, both therapeutically and by surgical intervention. Furthermore, the patient was subjected to a surgery called Commando, with a high risk of mortality. However, the patient survived for 5 years after the reintervention by surgery and received multidisciplinary management including antioxidants such as vitamin C. There was no follow-up study to evaluate the effects of the antioxidant therapy or the evolution and survival achieved after the critical period, but this confirms the findings of a large series carried out by our group, where OS was controlled through standard management and adjuvant therapy with antioxidants.

The third patient with IE was a 67-year-old man with a bicuspid aortic valve. A large series of patients with this condition has demonstrated that a high percentage of IE may be present in them [33]. In this case, *Streptococcus viridians* was found as the main pathogen, which is also one of the most frequently found bacteria. This patient has survived for 4 years so far, but, in his file, we did not find complete data on the therapeutic management that he received. Nevertheless, he was in an intensive therapy area, where standard management is always implemented, and there was an immediate surgical resolution (Supplementary Materials Video S1).

On the other hand, the CSs that were selected for comparison versus the patients with IE were subjected to AVR, and they underwent the same procedures as the patients with IE. Although they were not healthy subjects, and it was expected that they also presented alterations in redox homeostasis, the difference between the groups lay in the presence of IE. It should be noted that the subjects who were selected as controls and the patients with IE had different indications for aortic valve replacement, and this could be a topic of discussion regarding the loss of redox homeostasis. However, the point of comparison lies in the presence and absence of IE.

*Study Limitations*. While the small number of cases could be considered a limitation, our objective was to carry out an exploratory study on this topic to determine its feasibility for a posterior study, with this being a prospective study. Exploratory or preliminary studies are often recommended to evaluate in advance which findings deserve attention, without investing large amounts of resources. In this sense, there are studies on IE in large series of patients, but none have focused on elucidating the participation of OS independently of the origin and comorbidities of the patient.

## 5. Conclusions

The results from this series of three cases of IE in an aortic valve context suggest that the bacterial infection favors oxidative stress in the aortic valve, which leads to an increase in the activity of the SOD isoforms and peroxidases, and this contributes to the loss of the intricate redox homeostasis system in patients with IE. This causes a positive feedback oxidative background that leads to damage in the heart, likely resulting in a fatal outcome. The surgical management of IE in expert hands improves these complex conditions; however, the success of the intervention must be accompanied by follow-up during the pre- and post-surgical periods, aiming to reduce the metabolic conditions caused by the comorbidities in each patient. In addition to reducing the infectious and inflammatory state, the presence of the deregulation of OS requires the consideration of adjuvant management with antioxidant therapy. The findings also justify future studies through clinical trials, where an adequate sample size could be included to confirm and support this hypothesis.

*Study Advantages.* Despite the small number of cases, this is one of the few studies conducted on human valve tissue. The findings support the notion that OS is involved in the mechanism of damage of IE as an alternative mechanism that deserves attention, as well as the possible consideration of antioxidant therapeutic treatment. In this sense, an antioxidant therapy as an adjuvant could be added to the standard management in patients with IE to increase the TAC, represented mainly by GSH and thiol groups, resulting in an increase in the enzymatic activity of GPx and TxrR. This therapy could decrease the OS and contribute to lower valve aortic damage induced by bacterial endotoxins. In this regard, our research group has previously demonstrated that antioxidant therapy with vitamins C or E, melatonin or N-acetyl-cysteine in patients with septic shock and SARS-CoV-2 is effective against bacterial or viral infections, and it contributed to better survival among these patients [13,34].

## Figures and Tables

**Table 1:** General characteristics of the three patients with IE and of the CSs.

	Endocarditis (*n* = 3)	Control Subjects (*n* = 3)
Age (years)	70 (64–83)	65 (43–67)
Body mass index (weight/height^2^)	22 (20–25)	29 (28–33)
Comorbidities (*n* and %)		
Diabetes mellitus	1 (33)	1 (33)
Systemic arterial hypertension	2 (66)	2 (66)
Dyslipidemia	1 (33)	1 (33)
Smoking	2 (66)	2 (66)
Laboratory Parameters		
Glucose (mg/dL)	119 (85–131)	115 (109–164)
Creatinine (mg/dL)	0.81 (0.7–1.5)	1.4 (0.98–1.7)
Blood urea nitrogen (mg/dL)	30 (22–41)	18 (15–23)
Uric acid (mg/dL)	6.7 (3.8–7)	9.8 (5.3–10.1)
Natriuretic peptide NT-Pro-BNP (pg/mL)	7339 (1971–22,494)	200 (170–278)
Hemoglobin (g/dL)	11.8 (11.1–14.7)	14.4 (13.5–15.9)
Platelets (10^3^/µL)	172 (152–200)	160 (91–230)
Leukocytes (10^3^/µL)	8.4 (5.6–16.1)	8 (6.5–10)
Lymphocytes (10^3^/µL)	1.1 (1–8.7)	2.5 (1.2–2.7)
Neutrophils (10^3^/µL)	6.6 (4.5–13.9)	6.1 (3.1–7)
Erythrocyte sedimentation rate (mm/h)	30 (27–43)	18 (16–32)
C-reactive protein (mg/L)	47.5 (40–200)	2.5 (0.7–6.9)
Total cholesterol (mg/dL)	173 (138–325)	112 (108–186)
High-density lipoprotein (mg/dL)	53.4 (45.5–53.8)	44 (28.5–44.5)
Low-density lipoprotein (mg/dL)	98 (92–196)	122 (101–125)
Triglycerides (mg/dL)	131 (130–211)	2123 (70–221)
Aorta Diameters (mm)		
Aortic valve plane	29 (20–38)	26 (22–27)
Sinus of Valsalva	37 (20–40)	28 (21–58)
Sino tubular junction	38 (18–40)	27 (21–58)
Ascending aorta	37 (16–39)	30 (20–37)
Ejection fraction of the left ventricle (%)	20 (20–45)	44 (30–65)

Abbreviations: NT-Pro-BNP = natriuretic peptide.

**Table 2:** The demographic and diagnostic characteristics of the patients with IE and CSs.

Patients with IE						
Case	Age	G	BMI	Diagnosis	Evolution	Death
1	55	F	20	2009 diagnosis of Arteritis de Takayasu plus bicuspid aortic valve SAH 1999 AVR by severe AoI (Medtronic Hall).	Endocarditis in 2016. Univalve prosthetic valve with pannus and abscess with tissue destruction at level of interventricular septum with bacterial growth with *Staphylococcus aureus* and *epidermidis*. Surgery AVR and implantation of dual-chamber pacemaker by complete BAV; she had ischemic stroke and left hemiparesis with evolution with reduced heart failure LVEF 25% and died in 2017, one year after surgery.	yes
2	78	F	24	DAoI and stenosis AoI, trivalve aortic valve. In March 2019, she received intervention with TAVI. Portico 25 mm plus Boston Scientific DDD pacemaker, tachycardia–bradycardia syndrome. Comorbidities: smoking, systemic arterial hypertension and dyslipidemia.	In 2009, the patient was diagnosed with DAoI with predominant stenosis, which did not require surgery at that time. Four years later, the patient presented tachycardia–bradycardia syndrome and a Boston Scientific DDD permanent pacemaker was placed. In 2019, ten years after the initial DAoI diagnosis, the stenosis worsened and TAVI was considered for AVR. Five days later, the patient presented mitral–aortic endocarditis, which required an explant transcatheter implanted with a prosthetic aortic valve plus resection of the ascending aorta plus MVR with an Edwards Perimount 21 biological prosthesis plus Bentall and Bono surgery with a Woven Dacron 24 tube. In April 2019, the patient underwent TAVI and developed endocarditis with vegetation in the aortic valve. Mitral–aortic junction abscess plus aortitis aortic wall abscess. Valve crop *Enterobacter cloacae*, *Klebsiella oxytoca*. She was treated with aortic valve explanation surgery, ascending aorta resection. Bentall and Bo, MVR Edwards Peri mount. Mitroaortic command surgery. As of 2024, she is alive with LVEF 40%.	no
3	67	M	30	In 2019, DAoI and AoI severe bicuspid aortic valve, LV systolic and diastolic dysfunction LVEF 20%, smoking.	In 2020, native valve endocarditis with mobile vegetation of left non-coronary valve plus ascending aortic aneurysm, surgery AVR Medtronic Hall with bacterial growth with *Streptococcus viridans.* In 2024, normal functioning of prosthesis, LVEF 57%.	no
Control Subjects						
4	60	F	30	Ischemic heart disease, tri-valvular disease; 2014, angioplasty in right coronary artery; 2017, aortic stenosis, AVR with mechanical prosthesis St Jude Masters HP 21, LVEF 52%. Comorbidities: diabetes mellitus.	2024, asymptomatic LVEF 58%.	no
5	37	M	28	2018, ascending aortic aneurysm with aortic insufficiency, 4-cavity dilatation eccentric hypertrophy LV, severe mild mitral insufficiency, PAP 74 mmHg, surgery AVR, DM, systolic. Arterial hypertension, dyslipidemia, hyperuricemia, positive smoking. LVEF 44%.	2019, gout, asymptomatic cardiovascular LVEF 50%, systolic dysfunction GLS 14.5.	no
6	62	M	34	Ventricular dysfunction, severe aortic and mitral insufficiency, generalized hypokinesia, LVEF 30%. 2014, surgery due to aortic dissection, Stanford A, DeBakey 1 plus severe tricuspid regurgitation and mechanical AVR St. Jude. SAH, smoking.	2017, asymptomatic, controlled high blood pressure, stopped attending after this date.	no

Demographic characteristics of the three patients with IE that required aortic valve replacement surgery and control subjects with aortic valve damage who required aortic valve replacement and did not develop endocarditis and were included in the study. Abbreviations: DAoI = double aortic injury, AVR = aortic valve replacement, AoI = aortic insufficiency, Ao = aortic, LV = left ventricle, LVEF = left ventricle ejection fraction, TAVI = transcatheter aortic valve implantation, MVR = mitral valve replacement, GLS = global strain, BAV = atrioventricular block, PAP = pulmonary arterial pressure, DM = diabetes mellitus, SAH = systemic arterial hypertension.

## Data Availability

The datasets generated and analyzed during the current study are available from the corresponding author on reasonable request.

## Supplementary Materials

Following supporting information can be downloaded at: https://www.mdpi.com/article/10.3390/diagnostics14242807/s1, Video S1: Transesophageal echocardiographic analysis of the patient series. Case 1: Transgastric short axis showing vegetation (yellow arrow) in relation to the subvalvular mitral apparatus. Case 2: showing mitral valve endocarditis (yellow arrow) and severe mitral regurgitation. Case 3: Transesophageal short axis and three-chamber view, Also showing vegetation in the aortic valve (yellow arrow); Echocardiographic findings.
